# Widening Participation in Dentistry: A Quantitative Study of Changing Demographics at a South West UK Dental School (2014–2021)

**DOI:** 10.1111/eje.70131

**Published:** 2026-03-10

**Authors:** Rebecca Glanville, Marie Bryce, Sally Hanks, Ian Mills, Daniel Zahra

**Affiliations:** ^1^ Faculty of Health University of Plymouth Plymouth UK

**Keywords:** dental student demographics, dentistry recruitment and retention, undergraduate dental admissions, widening participation

## Abstract

**Introduction:**

Access to National Health Service (NHS) dental care in the UK, particularly in rural and coastal regions such as the South West, remains a significant concern. Increasing socio‐economic diversity in the dental workforce has previously been reported to improve access to care in underserved areas. Widening Participation (WP) initiatives aim to address this by encouraging applications from underrepresented groups.

**Methods:**

This study analysed admissions data from the University of Plymouth's Bachelor of Dental Surgery (BDS) programme for cohorts between 2014 and 2021. Metrics included entry qualifications, age, gender, ethnicity, disability and socio‐economic markers. Statistical analyses included chi‐squared tests and ANOVA to identify demographic shifts over time.

**Results:**

Data from 512 students revealed significant changes in entry profile *(p* < 0.001), gender distribution (*p* = 0.003), school type (*p* = 0.003) and age at entry (*p* < 0.001). The proportion of students applying with predicted grades increased, while graduate applicants declined. Socio‐economic diversity remained limited, with most students from areas of high participation in higher education and low deprivation.

**Conclusion:**

Over the study period, the demographic profile of dental students remained largely unchanged, even though this period coincided with enhancements to widening participation initiatives. Without greater representation from diverse backgrounds, the dental workforce may struggle to meet the needs of underserved communities.

## 1 Introduction

The shortfall in provision of care by National Health Service (NHS) dentists in the United Kingdom, and particularly in areas of ‘dental desert’ like the South West, is of tremendous national concern [1]. Lack of NHS dentistry, particularly in remote, rural or coastal areas is reported as a crucial factor in the increase in oral disease [2]. A 2021 oral health needs assessment of the South West region identified extensive waiting lists, a falling percentage of children attending an NHS dentist and predicted population growth [3]. Studies have shown increasing numbers of dentists leaving the NHS and the profession [4], exacerbating the shortage of NHS dentists, further reducing access to treatment and increasing the workload and stress for practitioners. Remaining dentists feel driven to ‘perform’ and that they are in a ‘survival culture’ thereby increasing the likelihood of attrition [5].

The current lack of socio‐economic diversity in the workforce compounds the issue of dental deserts. James et al. [6] have demonstrated that graduating healthcare practitioners are more likely to practice in a community with a similar socio‐economic background to their own. Girotti et al. [7] found that increased diversity of clinicians can encourage increased access among patients. While not focussed on dentists, Dowell et al. [8] found that General Medical Practitioners had a 4.3 times higher likelihood of working in a deprived area if they were from a lower socio‐economic background with parents working in non‐professional roles. Additionally, those graduates with a background in a remote, rural or coastal area were more likely to return to these environments to practice [8].

There have been national strategies for increasing diversity of the NHS workforce and, separately, schemes to enable greater participation in higher education across all sectors since 2014 [9]. In spite of this, however, students from lower socio‐economic groups or non‐traditional backgrounds continue to be significantly underrepresented in undergraduate clinical degree programmes, including dentistry [10].

The Universities and Colleges Admissions Service (UCAS) facilitates and collates applications, offers and offer acceptances for undergraduate (and some postgraduate) university courses in the United Kingdom (UK). UCAS also offers a tariff system where academic qualifications and some extracurricular activities are converted into tariff points used by some universities as admission criteria. Current admissions processes predominately focus on academic qualifications and UCAS tariff, with students able to gain additional tariff points from high‐level extracurricular activities (e.g., music or drama examinations). The costs associated with these activities are likely to preclude students from lower socio‐economic backgrounds from gaining these additional UCAS tariff points. As such, this may dissuade or disadvantage students from a more diverse range of backgrounds applying to University [11]. Ensuring that a career in dentistry is accessible to a wider range of students offers benefits to social equality (social justice and equal opportunities for all) and also provides clinicians who are more understanding of their practice culture and community, and better able to communicate and empathise with their patients [12].

In 2015, the UK government set targets to increase the proportion of students from disadvantaged and other underrepresented backgrounds progressing to higher education [13]. Widening participation initiatives at the University of Plymouth were expanded and formalised from 2014 onwards in response to the national strategy changes and were offered to students meeting contextual markers (including being a care leaver, receiving free school meals or being resident in an area of socio‐economic deprivation). During the study period, additional pathway activities were introduced or strengthened, including enhanced work‐experience opportunities, mentoring, summer schools and pathway workshops [14]. These initiatives were delivered consistently throughout the study period, with incremental expansion rather than a single implementation point. Widening Participation programmes were not designed as a recruitment tool for the University of Plymouth, or to reduce the rural–urban divide but to reduce barriers to higher education for underrepresented groups.

This study aims to identify whether these enhancements of WP initiatives have increased the number of students from WP backgrounds enrolling on the Bachelor of Dental Surgery (BDS) programme at the University of Plymouth.

## 2 Materials and Methods

Historic admissions and demographic data were collated from the 2014 to the 2021 student intakes to the University of Plymouth 5‐year undergraduate Bachelor of Dental Surgery (BDS) programme inclusively. These cohorts were selected to ensure comparable entry requirements. The 2020 and 2021 entry intakes were impacted by the Covid‐19 pandemic and students were awarded ‘centre assessed grades’ based on modified teacher predictions rather than examination results. The impacts of the Covid‐19 intakes will be discussed further later in this paper.

The metrics collected can be found in Table 1.

**TABLE 1:** Variable definitions and category descriptions.

Variable	Categories	Definition
Type of entrant	Direct school leaver	The student applied within 2 years of completing tertiary education with predicted or achieved grades, or while taking resit Level 3 qualifications
	Graduate/GAMSAT	The student applied more than 2 years after tertiary education, as a graduate or without the standard academic qualifications by using the Graduate Medical School Admissions Test (GAMSAT) in lieu of academic qualifications
	SoBHS Graduate	The student applied with a first‐class degree from the University of Plymouth School of Biomedical and Healthcare Sciences
	SoBHS Transfer	The student applied to transfer from the first year of an undergraduate programme within the University of Plymouth School of Biomedical and Healthcare Sciences
Age	NA	In years at point of entry
Gender	Female/Male/Other	Self‐disclosed at point of entry
Ethnicity	Asian/Other ethnicity/White	Self‐disclosed at point of entry and grouped to ensure anonymity and provide sufficient data points per group for comparison
Disability	No known disability/Specific learning difficulty/Other disability	Self‐disclosed at point of entry and grouped to ensure anonymity and provide sufficient data points per group for comparison
POLAR 4	NA	Participation of Local Areas measure—a measure of the level of participation in higher education by postcode. Scale from 1 to 5 with 1 being lowest level of participation and 5 being highest level of participation in higher education. This value was only available for students with a home address in the United Kingdom
Index of Multiple Deprivation (IMD)	NA	A measure of the level of deprivation in an area by postcode. Scale from 1 to 10 with 1 being most deprived area and 10 being least deprived area. This value was only available for students with a home address in England
School type	College	A non‐fee paying, non‐selective sixth‐form or further education college specialising in Level 3 qualifications
	Comprehensive	A non‐fee paying, non‐selective school for students aged between 11 and 16 or 11 and 18
	Independent	A fee‐paying, private school for students aged up to 16 or 18
	Selective	A non‐fee‐paying selective grammar school for students aged between 11 and 16 or 11 and 18
	Undisclosed/not applicable	Information not available or student has applied as a graduate or with GAMSAT

Results were tabulated and tested for normality. All analyses were completed using R version 4.0.5 within RStudio version 2023.06.2.

Significant variation in categorical variables (type of entrant, gender, ethnicity, type of school) was tested using Chi‐squared tests for independence. These analyses were conducted using the chisq.test() function in base R using Monte Carlo simulation of *p*‐values (simulate.p.value = TRUE, B = 100 000) to circumvent any problems in the model caused by small values. The contingency tables used for analysis contained the full, underlying data (although categories with fewer than three students are masked in Table 2 to preserve anonymity complete counts were included in the statistical analyses). Table dimensions therefore reflect the original structure of each variable.

**TABLE 2:** Entrant profile summary.

Number of entrants	2014 entry	2015 entry	2016 entry	2017 entry	2018 entry	2019 entry	2020 entry	2021 entry	All
	57	55	58	58	59	61	82	82	512
Type of entry									
DSL^a^ achieved	12 (21.05%)	9 (16.36%)	17 (29.31%)	5 (8.62%)	4 (6.78%)	9 (14.75%)	6 (7.13%)	10 (12.20%)	72 (14.06%)
DSL^a^ predicted	20 (35.09%)	30 (54.56%)	22 (37.93%)	37 (63.79%)	39 (66.10%)	40 (65.57%)	62 (75.61%)	59 (71.95%)	309 (60.35%)
DSL^a^ resit	14 (24.56%)	12 (21.82%)	9 (15.52%)	9 (15.52%)	8 (13.56%)	5 (8.20%)	11 (13.41%)	8 (9.76%)	76 (14.84%)
Graduate/GAMSAT^b^	11 (19.30%)	< 3	8 (13.79%)	4 (6.90%)	6 (10.17%)	5 (8.20%)	3 (3.66%)	< 3	40 (7.81%)
SoBHS^c^ Graduate	< 3	< 3	< 3	< 3	< 3	< 3	< 3	< 3	9 (1.76%)
SoBHS^d^ Year 1 transfer	< 3	< 3	< 3	< 3	< 3	< 3	< 3	< 3	6 (1.17%)
Age at entry									
Max	40	25	27	29	40	33	28	22	17
Min	17	18	17	18	18	18	18	18	40
Median	19	19	19	18	18	18	18	18	18
Mean	20.53	19.16	19.47	19.24	19.53	19.16	18.68	18.68	19.24
Gender									
Female	32 (56.14%)	30 (54.55%)	25 (43.10%)	31 (53.45%)	28 (47.46%)	34 (55.74%)	61 (74.39%)	56 (68.29%)	297 (58.01%)
Male	25 (43.86%)	25 (45.45%)	33 (56.90%)	27 (46.55%)	31 (52.54%)	27 (44.26%)	21 (25.61%)	26 (31.71%)	215 (41.99%)
Ethnicity									
Asian	24 (42.11%)	26 (47.27%)	26 (44.83%)	22 (37.93%)	31 (52.54%)	27 (44.26%)	35 (42.68%)	42 (51.22%)	233 (45.51%)
Other ethnicity	5 (8.77%)	8 (14.55%)	11 (18.97%)	9 (15.52%)	9 (15.25%)	8 (13.11%)	17 (20.73%)	13 (15.85%)	80 (15.62%)
White	26 (45.61%)	21 (38.18%)	18 (31.03%)	26 (44.83%)	18 (30.51%)	26 (42.62%)	29 (35.37%)	26 (31.71%)	190 (37.11%)
Undisclosed	< 3	< 3	3 (5.17%)	< 3	< 3	< 3	< 3	< 3	9 (1.76%)
Disability									
No disability	50 (87.72%)	53 (96.36%)	47 (81.03%)	53 (91.38%)	57 (96.61%)	54 (88.52%)	74 (90.24%)	78 (95.12%)	466 (91.02%)
Other disability	3 (5.26%)	< 3	4 (6.90%)	< 3	< 3	< 3	3 (3.66%)	< 3	16 (3.13%)
Specific learning difficulty	4 (7.02%)	< 3	7 (12.07%)	4 (6.90%)	< 3	5 (8.20%)	5 (6.10%)	3 (3.66%)	30 (5.86%)
POLAR 4									
Max	5	5	5	5	5	5	5	5	5
Min	1	1	1	1	1	1	1	1	1
Median	4	4	5	4	4	4	4	4	4
Mean	3.61	3.79	4.20	3.46	3.98	3.70	3.84	4.04	3.83
IMD^e^									
Max	10	10	10	10	10	10	10	10	10
Min	1	1	1	1	1	1	1	1	1
Median	7	5	7	5	6	8	7	7	7
Mean	6.57	5.84	6.17	5.74	5.94	7.04	6.46	6.33	6.31
School type									
College (state)	6 (10.53%)	7 (12.73%)	7 (12.07%)	11 (18.97%)	13 (22.03%)	11 (18.03%)	8 (9.76%)	11 (13.41%)	74 (14.45%)
Comprehensive (state)	12 (21.05%)	14 (25.45%)	9 (15.52%)	20 (34.48%)	14 (23.73%)	21 (34.43%)	21 (25.61%)	23 (28.05%)	134 (26.17%)
Independent	9 (15.79%)	12 (21.82%)	14 (24.14%)	8 (13.79%)	8 (13.56%)	8 (13.11%)	23 (28.05%)	32 (39.02%)	114 (22.27%)
Selective (state)	14 (24.56%)	13 (23.64%)	16 (27.59%)	12 (20.69%)	13 (22.03%)	12 (19.67%)	24 (29.27%)	10 (12.20%)	114 (22.27%)
Undisclosed/not applicable	16 (28.07%)	9 (16.36%)	12 (20.69%)	7 (12.07%)	11 (18.64%)	9 (14.75%)	6 (7.32%)	6 (7.32%)	76 (14.84%)

*Note:* Measures: Age—age in years as at the first of September during the year of entry; Gender—self‐reported gender at point of application; Ethnicity—self‐reported ethnicity at point of application; Disability—self‐reported disability at point of application; POLAR 4—Participation of Local Areas measure—a measure of the level of participation in higher education by postcode. Scale from 1 to 5 with 1 being the least level of participation and 5 being the highest level of participation. This value is only available for students with a home address in the United Kingdom; School type—obtained from OFSTED records or school websites.

^a^ DSL—direct school leaver—the candidate applied within 2 years of completing tertiary education. These students can apply with all Level 3 qualifications achieved, all Level 3 qualifications pending (predicted), or with the qualifications achieved but at a lower level than required (resit).

^b^ Graduate/GAMSAT—applicants more than 2 years after tertiary education, applying with a degree or without standard academic qualifications. These students can take the Graduate Medical School Admissions Test aptitude test in lieu of academic qualifications.

^c^ SoBHS Graduate—the candidate applied with a first‐class degree from the University of Plymouth School of Biomedical and Healthcare Sciences.

^d^ SoBHS Transfer—the candidate applied to transfer from the first year of a programme at the University of Plymouth School of Biomedical and Healthcare Sciences.

^e^ IMD—Index of Multiple Deprivation—a measure of the level of deprivation in an area by postcode. Scale from 1 to 10 with 1 being most deprived and 10 being least deprived. This value is only available for students with a home address in England.

Following confirmation of the normal distribution of the data using Shapiro–Wilk tests, ANOVA (Analysis of Variance) was employed to identify any statistically significant variation between the year of entry and the continuous variables (age at point of entry, Participation of Local Areas [POLAR 4] and Index of Multiple Deprivation [IMD] decile).

Post hoc comparisons were not conducted as the objective was to assess overall variation in demographic profiles across the study period rather than differences between specific years or categories.

## 3 Results

Following collation of historic admissions data, 512 student profiles were identified. A breakdown of student profiles by year of entry can be found in Table 2. Where there are fewer than three students within a group, the exact values have been hidden to preserve anonymity.

The results of the statistical analyses can be found in Table 3. It can be seen that, across the eight cohorts included in the analyses, there have been significant changes in student profile across type of entry, gender, school type and age.

**TABLE 3:** Demographic analyses—independent variable: year of entry.

Type of analysis	Variable	*X* ^*2*^	*p*
Chi‐squared	Type of entry	77.452	< 0.001***
Chi‐squared	Gender	21.557	0.003**
Chi‐squared	Ethnicity	10.574	0.724
Chi‐squared	Disability	15.423	0.347
Chi‐squared	School type	53.338	0.003**
		**Sum of sq**	***p***
ANOVA	Age at point of entry	81.965	< 0.001***
ANOVA	POLAR 4	9.513	0.191
ANOVA	IMD	1.766	0.575

*Note:* * indicates statistically significant to *p* < 0.05. ** indicates highly significant to *p* < 0.01. *** indicates very highly significant to *p* < 0.001.

### 3.1 Type of Entry

In the UK, most school‐leaver applicants apply for university before receiving their final A‐level results and therefore submit their applications with grade predictions provided by their school or college rather than achieved grades. At the University of Plymouth, ‘direct school leavers’ are defined as applicants applying within 5 years of completing their A‐levels and presenting valid Level 3 qualifications. Consequently, direct school leavers may apply with either predicted or achieved grades depending on their time since school completion. Students applying with predicted grades are made an offer conditional on their achieving a certain grade profile and can be accepted or rejected once the final results are received.

The proportion of students successfully enrolling having applied with predicted A‐level grades has increased throughout the period sampled (*X* ^2^ = 77.452, ***p < 0.001). The number of graduates applying has decreased. One possible explanation is the introduction in 2015 of a guaranteed interview scheme for graduates from the University of Plymouth School of Biomedical and Healthcare Sciences. Under this scheme, students with a predicted or achieved first‐class degree are offered a guaranteed interview for the dentistry programme, bypassing the normal selection process, separating these students from external graduate applicants. To check for any impact of the Covid‐19 pandemic on the student demographic the 2020 and 2021 cohorts were removed from the analysis. In these two cohorts, rather than take summative examinations, students were awarded Centre‐Assessed Grades (CAGs) by their school or college. Overall, these CAGs were typically higher than a similar cohort would achieve through examination [15], However, removal of these cohorts still resulted in significant variation by type (*p = 0.016).

### 3.2 Gender

While the gender balance of students remained reasonably consistent from the 2014 to 2019 years, there was a significant increase in the proportion of female students during the 2020 and 2021 entry cycles (**p = 0.003). The significant variation by gender is not present if these cohorts are excluded (*p* = 0.666).

### 3.3 Ethnicity

The ratio of student ethnicities has remained reasonably consistent, with the largest proportions of students identifying as having a White or Asian background.

### 3.4 Disability

The proportion of students reporting disabilities has remained reasonably consistent, with the majority of students declaring ‘no known disability’.

### 3.5 School Type

The proportion of students from independent (fee‐paying) schools has increased across time while the number of students from state‐funded selective schools has decreased (**p = 0.003). The proportion of students from state‐funded non‐selective schools (comprehensive schools) has been variable, but statistically not significant. The majority of students with an ‘undisclosed’ school background (in green) were applying as graduates and so the proportion has reduced in line with the reduction of graduate‐entry applicants. When the 2020 and 2021 cohorts were removed, the overall association was no longer statistically significant (*p* = 0.458).

### 3.6 Age at Point of Entry

The average age of students at point of entry has shown statistically significant variation (***p < 0.001). These results may have been skewed by the students in their thirties and forties acting as outliers in the 2014 and 2018 entry cycles. The significant changes to the age of applicants are again not present if the 2020 and 2021 cohorts (which contained a higher proportion of younger students) are excluded (*p =* 0.071).

### 3.7 POLAR 4

The distribution of POLAR 4 quintiles has remained consistent with a mean around quintile 4. This indicates that the cohort demographics are skewed towards students from areas with increased levels of participation in Higher Education.

### 3.8 IMD

The distribution of IMD deciles has remained consistent with means between decile 5 and 7. This indicates that the cohort demographics are skewed towards students with lower levels of socio‐economic deprivation.

## 4 Discussion

The analyses identified specific changes in the profile of dental cohorts admitted between 2014 and 2021 with significant changes to the type of entry (***p < 0.001), type of school attended (**p = 0.003), student gender (**p = 0.003) and student age on entry (***p < 0.001).

### 4.1 Type of Entry

Academic entry profile changes are evidenced by an increase in the proportion of successful applicants holding pending or predicted Level 3 qualifications at the point of entry rather than applying following a gap year or with achieved or resit qualifications. This suggests that fewer applicants are taking gap years and instead applying to enter university directly from school. These findings are in contrast with national studies showing an increase in deferred entry to university during and immediately following the Covid‐19 pandemic [16]. While students may wish to progress on their career trajectory, taking a gap year can improve a student's interpersonal and professional skills and academic motivation [17, 18].

The data show a corresponding decrease in candidates applying while resitting previously completed qualifications in the hope of improving their outcomes. This is particularly observable in the 2020 and 2021 cohorts, and most likely a reflection of the use of Centre Assessed Grades (CAGs) used as mitigation in the Covid‐19 pandemic. Fewer students failed to achieve their UCAS offer and so were accepted with their first‐attempt grades. This may also have impacted the following (2022) cohort as there was a smaller proportion of students needing to apply while taking resits.

While this would seem an obvious reason for the variation, excluding the 2020 and 2021 cohorts shows a similar pattern of increasing proportions of successful applicants having applied while holding predicted grades. As such, this variation cannot be attributed entirely to the Covid‐19 assessment modifications.

The increase in students applying with predicted qualifications may impact cohort profiles. McManus et al. [19] identified that teacher‐provided A‐level predictions were over‐predicted in almost half (44.7%) of cases and have lower predictive validity for subsequent academic performance than achieved grades. As such, applicants with under‐predicted grades may be at a disadvantage during the admissions process and rejected in favour of their peers with non‐representative over‐predicted grades.

There has also been speculation that predicted grades may favour or disadvantage certain student groups, with studies identifying gender variations [20, 21], variation by ethnicity [22] and variation by socio‐economic status [23]. A meta‐analysis found no evidence of systematic bias against any applicant group [24] but these publications have a number of confounding factors or limitations and so further investigation in this area would be beneficial.

### 4.2 Type of School Attended

The significant variation in type of school attended can potentially be another factor impacted by the Covid‐19 pandemic and CAGs with studies showing that independent schools are more likely to over‐predict A‐level grades [23]. Additionally, students at independent schools benefited disproportionately from CAGs, with a larger increase in the proportion of A or A* grades compared to most other types of schools [25].

In spite of the lack of significant variation in school type when the 2020 and 2021 cohorts are excluded, the trend does seem to show an increase in students from independent schools when compared to the rest of the cohort and, with increases in WP activities, it would be expected that the proportion of students from state and comprehensive schools would increase.

Studies have shown that 13% of students from independent schools achieve top A‐level grades needed for clinical university programmes (defined as AAB) while only 2% of students with free school meals (a frequently used WP marker) receive these grades [26]. As such, initial filtering of applications by academic grade may reduce the proportion of students from a WP background progressing to further stages of the admissions process.

Overall, 22.3% of students enrolling on the undergraduate BDS programme at the University of Plymouth have attended independent schools. This is significantly higher than the 6.1% of British school‐age children attending fee‐paying schools [27]. Selective schools have also been shown to disproportionately recruit students from higher socio‐economic backgrounds [28, 29]. With 52% of the enrolled BDS students being from fee‐paying or grammar schools (excluding those with undisclosed or not applicable school backgrounds), it can be seen that the educational background of the students is skewed towards those from higher socio‐economic groups.

There seems to be an increasing proportion of successful applicants originating from independent schools, and a lower proportion of students from areas with high deprivation or low education participation rates. This suggests that, while WP initiatives may be increasing the diversity of the applicant pool, failure to make adjustments to the student selection processes—including the continued reliance on predicted A‐level grades—has resulted in the current profile of the dental students mirroring that from a decade earlier [30]. This lack of improvement of the socio‐economic and educational background diversity of dental students, despite various WP initiatives, has also been confirmed by other studies [11].

### 4.3 Gender

Our results suggested that more young, female applicants enrolled during the 2 years in which Centre Assessed Grades were awarded. Following the conclusion of the Covid‐19 qualification cycles, a review of GCSE (a level 2 qualification typically taken at age 16) CAGs by Magowan found that there was evidence of positive bias by teachers towards females and, when intersectionality was considered, female students of medium prior attainment and higher socio‐economic backgrounds were more likely to be benefitted by teacher biases in the CAG process [31]. While this paper focused on GCSE‐level qualifications, this could be a reason for the increase in female entrants during the Covid‐19 intakes.

### 4.4 Age

The increase in younger students during the Covid‐19 intakes can also be attributed to the increased number of students accepted due to the CAGs and the reduction in the proportion of students applying following a gap year or with resit grades. While gap year or resit applicants are typically 19 or 20 years old, those entering university straight from school are generally 18, contributing to a younger overall intake.

### 4.5 POLAR 4 and IMD

In spite of WP initiatives across schools and universities, the POLAR 4 and IMD backgrounds of the entrants are still skewed towards more traditionally well‐represented groups.

While a fair and inclusive admissions process is essential to encouraging applicants from a wide range of backgrounds, the findings presented here suggest that current WP initiatives may not be delivering the intended outcomes. However, this does not necessarily mean WP has failed. Instead, it highlights the need to understand the broader context of higher education—particularly the impact of national and global challenges such as student debt, the rising cost of living, economic instability and the aftermath of Covid‐19. These wider pressures may disproportionately affect students from WP backgrounds, undermining the effectiveness of such initiatives. Further research is needed to explore these intersecting factors and to identify how admissions and support strategies can be strengthened to promote genuine equity in dental education.

## 5 Study Limitations

This study is based on admissions data from successfully enrolled students at a single institution and does not include applicant‐level data. This limits our ability to identify whether the trends identified reflect changes in selected students, applicant pool or the wider sector. Future research could explore these areas by further analysis of demographics at the point of application, including survival analysis to identify where students from WP backgrounds are more likely to be rejected. Multi‐institutional studies could also help identify whether the identified patterns reflect national trends.

## 6 Conclusion

This study found that, although the study period overlapped with an expansion of widening participation initiatives, the demographic profile of students enrolling onto the BDS programme remained broadly similar across cohorts, with students still disproportionately drawn from areas of low deprivation and from fee‐paying, independent schools.

While some of the issues may resolve naturally over time as the effects of the Covid‐19 pandemic and Centre Assessed Grades diminish, relying solely on this would be insufficient as some inequalities were still present when these cohorts were excluded.

If future cohorts continue to lack representation from the wider population (defined here as one that more closely reflects the socio‐economic and educational distribution of the general UK school‐age population), the dental workforce is unlikely to evolve in ways that effectively tackle existing inequalities and barriers to care. While a more diverse workforce alone cannot solve all the challenges related to dental access, increasing diversity through a more inclusive student intake is an important and necessary step towards improving equity and enhancing the profession's cultural competence.

## Author Contributions

Rebecca Glanville led the data collection, data analysis and drafting of the manuscript. Marie Bryce, Sally Hanks, Ian Mills and Daniel Zahra contributed to the development of the research methodology and narrative, provided critical revisions and offered supervisory and advisory support throughout. All authors reviewed and approved the final manuscript.

## Funding

The authors have nothing to report.

## Ethics Statement

Institutional ethical approval was obtained from the University of Plymouth, Faculty of Health Research Ethics and Integrity Committee on 11th December 2023 (Project Id: 3933).

## Conflicts of Interest

The authors declare no conflicts of interest.

## Data Availability

The data analysed in this study are not publicly available due to privacy and ethical restrictions. Requests for access to anonymised data may be considered by the corresponding author. Data are located in secured data‐storage with the University of Plymouth.
